# An Unusual Presentation of Isolated Leptomeningeal Disease in Carcinoma of Unknown Primary With Pancreatic Features

**DOI:** 10.1177/2324709613494830

**Published:** 2013-06-18

**Authors:** Madhurima Anne, Nazish Ahmad, Paul Lee, Mohamed Aziz, Yehuda Lebowicz

**Affiliations:** 1 Hofstra North Shore–Long Island Jewish School of Medicine, New Hyde Park, NY, USA; 2 Nassau Hematology Oncology, New Hyde Park, NY, USA

**Keywords:** carinomatous meningitis, leptomeningeal disease, carcinoma of unknown primary, pancreatic cancer

## Abstract

Leptomeningeal disease (LMD) can occur in a small percentage of patients with active metastatic cancer. However, we report a case of LMD occurring during disease remission in a patient with carcinoma of unknown primary with panreaticobiliary features. A 45-year-old woman was found with mediastinal and abdominal lymphadenopathy with lymph node biopsy consistent with adenocarcinoma, expressing immunomarkers CK7, CK20, and Ca19-9 along with markedly elevated serum Ca19-9 level. The patient was started on a pancreatic cancer directed chemotherapy regimen of Folfirinox (5-fluorouracil, leucovorin, oxaliplatin, irinotecan) and achieved complete response. She was then noted to have slowly rising Ca19-9 level that did not correlate with her lack of evidence of systemic disease progression. Eventually, she presented with neurologic symptoms and was found on imaging to have isolated LMD.

## Introduction

Leptomeningeal disease (LMD) or carcinomatous meningitis is a known entity that occurs in a small percentage of patients with metastatic cancer. It has been described mostly in the setting of breast and lung cancers. Other cancers which can rarely be causative include lymphoma, leukemia, melanoma, gastrointestinal cancers, as well as adenocarcinoma of unknown primary.^1-3^ It is unusual for solid tumors to manifest with LMD in the absence of systemic evidence of disease. We report a case of isolated leptomeningeal disease in a patient with carcinoma of unknown primary with features of pancreatic cancer who achieved systemic complete response after treatment with Folfirinox (5-fluorouracil, leucovorin, oxaliplatin, irinotecan) chemotherapy.

## Case

A 45-year-old woman with no significant past medical history was brought to our emergency department by her family members for shortness of breath and left calf pain. She underwent evaluation, including computed tomography (CT) of the chest with contrast, revealing pulmonary emboli within the right middle lobe segmental arteries, as well as mediastinal, hilar, and upper abdominal lymphadenopathy. The patient underwent further imaging, including CT of the abdomen with contrast, which revealed peritoneal implants, retroperitoneal and gastrohepatic lymphadenopathy, without evidence of possible primary source of malignant disease. The patient underwent a CT-guided biopsy of a periaortic lymph node, with pathology consistent with adenocarcinoma, expressing immunomarkers CK7, CK20, Ca19-9, and negative for TTF1, CDX2, CA125 and WT1. Magnetic resonance cholangiopancreatography (MRCP) done to ascertain a possible pancreatic source of disease was unrevealing. The patient was diagnosed with metastatic adenocarcinoma of unknown primary.

We were consulted at this time for management. Given the pathologic description, results of immunohistochemical staining as well as patient’s elevated initial serum Ca19-9 of 1040 U/mL (reference range <41.3 U/mL), we initiated treatment with a pancreatic cancer chemotherapy regimen of Folfirinox. Within 3 months, the patient achieved complete response by radiographic imaging with undetectable Ca19-9 level for 5 months.

**Figure 1.**
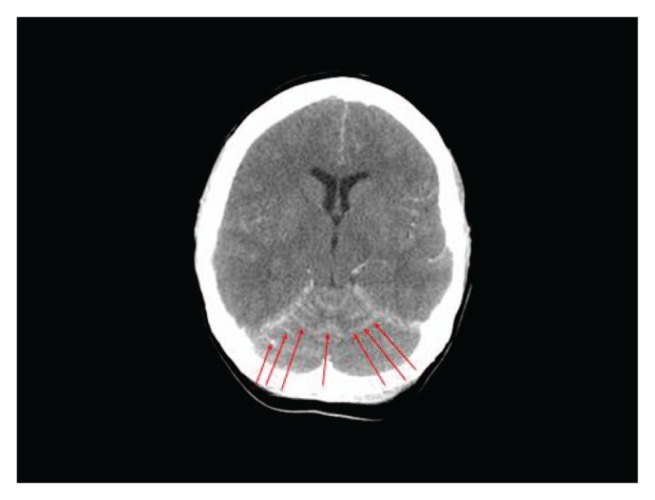
Axial postcontrast head computed tomogram demonstrating diffuse leptomeningeal enhancement involving both cerebellar hemispheres and the superior cerebellar vermis, as indicated by the red arrows.

**Figure 2.**
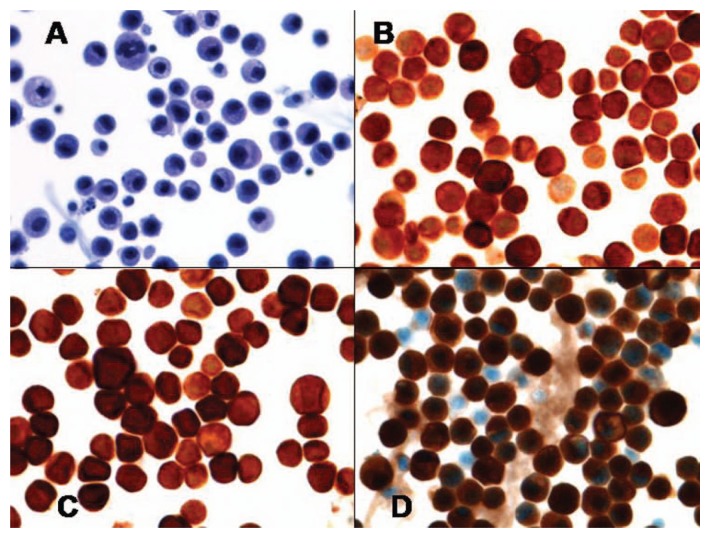
(A) Pap stain of cells from cerebrospinal fluid showing pleomorphic cells with nuclear atypia and abnormal mitosis. (B) Immunohistochemical stain of CK7 showing positivity in neoplastic cells. (C) Immunohistochemical stain of CK20 showing positivity in neoplastic cells. (D) Immunohistochemical stain of Ca19-9 showing positivity in neoplastic cells.

**Figure 3.**
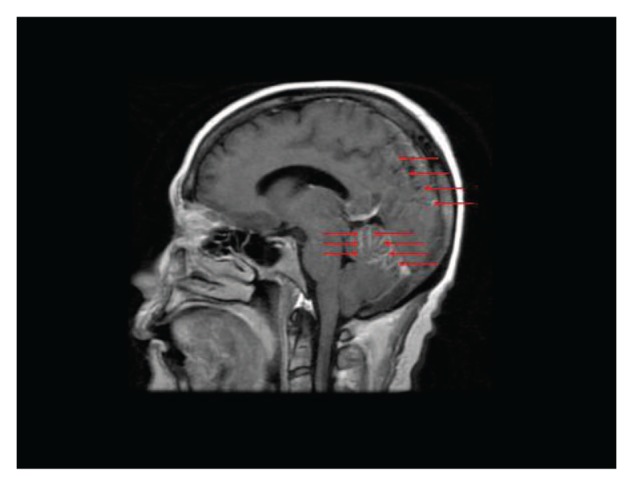
Saggital T1-weighted postcontrast magnetic resonance image of brain demonstrating diffuse leptomeningeal enhancement involving both cerebellar hemispheres, the superior cerebellar vermis, and parieto-occipital lobes, as indicated by the red arrows.

**Figure 4.**
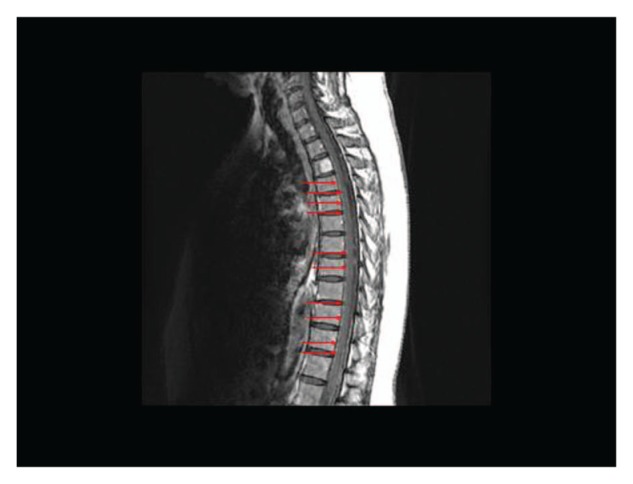
Saggital T1-weighted postcontrast magnetic resonance image of lower cervical and thoracic spine demonstrating subtle diffuse leptomeningeal enhancement of the spinal cord, as indicated by the red arrows.

She was subsequently noted to have a slowly rising Ca19-9 level, but without any symptoms or evidence of systemic disease progression on repeat radiographic imaging. After 12 months from initial diagnosis, the patient was brought to the emergency department with sudden onset of headaches, slurred speech, generalized weakness, and agitation. At this point, the Ca19-9 level was 1920 U/mL. Initial CT of the head with and without contrast showed evidence of leptomeningeal enhancement involving the posterior fossa (Figure 1). Analysis of cerebrospinal fluid (CSF) obtained via lumbar puncture revealed mildly elevated protein level of 50 mg/dL (reference range = 15-45 mg/dL), with normal glucose level of 67 mg/dL (reference range = 40-70 mg/dL). Cytological examination of CSF demonstrated malignant cells that were morphologically and immunohistochemically identical to the original biopsy from the periaortic lymph node metastasis (Figure 2). Magnetic resonance imaging of the brain and spine was consistent with abnormal leptomeningeal enhancement involving bilateral cerebellar hemispheres, vermis, parieto-occipital lobes, as well as diffuse enhancement of the spine (Figures 3 and 4). Treatment with intrathecal chemotherapy was discussed; however the family opted for palliative options and the patient rapidly deteriorated and expired.

## Discussion

We report an unusual sequela of LMD in carcinoma of unknown primary with features of pancreatic carcinoma. We treated the patient with a pancreatic cancer regimen of Fofirinox chemotherapy, and the patient achieved good systemic disease control for up to 1 year after the initial diagnosis. She developed LMD without any preceding symptoms, physical exam signs, or systemic evidence of disease progression on radiographic imaging, accompanied only by rising Ca19-9 level. Although the American Society of Clinical Oncology recommends against the routine use of Ca19-9 levels for establishing diagnosis for pancreatic cancer, it does state that levels can be measured at the start of treatment and if elevated, then followed every 1 to 3 months during treatment. It also establishes that rising Ca19-9 levels may be a marker for progressive disease requiring confirmation with other studies.^4^ In our case, Ca19-9 proved to be a reliable marker of disease activity.

Leptomeningeal disease resulting from solid tumors occurs generally in the setting of progressive systemic metastatic disease. Retrospective reviews reveal that whereas hematologic malignancies such as leukemia and lymphoma frequently present with LMD during periods of remission, about 80% to 90% of solid tumors have evidence of systemic disease at the time of diagnosis of LMD.^5,6^ The incidence of leptomeningeal involvement of gastrointestinal tumors and adenocarcinoma of unknown primary is very infrequent and has been reported in the range of 4% to 14% and 1% to 7%, respectively.^2,7^ Gastrointestinal cancers such as pancreatic and biliary cancers are aggressive diseases; hence, the majority of patients may succumb to progression of their systemic disease prior to developing and exhibiting signs of central nervous system metastases. Thus, a higher rate of brain metastases from pancreatic cancer has been discovered post mortem in comparison to the actual reported incidence of brain metastases from pancreatic cancer.^8,9^

Clinical manifestations of LMD can be vast and depend on degree of involvement of cerebral hemispheres, cranial and peripheral nerve roots, as well as spinal cord.^10^ Common symptoms of LMD include headaches, mental status changes, nausea, vomiting, gait difficulty, weakness, seizures, and back pain.^1,2,6^ Different cancer types have different mechanisms by which malignant cells enter the meninges. Hematologic dissemination is more common in hematologic than in solid malignancies. Breast and lung cancers can spread to the meninges via endoneural, perineural or perivascular lymphatic routes. Tumors located within the central nervous system parenchyma may spread to the meninges though direct spread. Another entity that has been described is iatrogenic spread during neurosurgical or other invasive procedures. Malignant cells that enter the meninges can then spread through the CSF by flowing to distant parts where they can form secondary leptomeningeal deposits. The pattern of these deposits also depends on the type of tumor. Diffuse leptomeningeal spread is characteristic of hematologic malignancies while plaque-like deposits is more typical for solid tumors.^2^

When LMD is suspected, radiographic imaging with T1-weighted gadolinium-enhanced magnetic resonance imaging of the brain and spine should be performed. Analysis of cerebrospinal fluid should be performed and classically reveals a high opening pressure (approximately 50% of patients), low glucose level, high protein level, and malignant cells on cytological examination. Elevated lactic acid dehydrogenase is a nonspecific marker, but may be noted to be elevated in the CSF of patients with LMD. Cytological evidence of malignant cells is necessary for establishing definitive diagnosis.^6,11^ Initial cytological analysis can be negative in up to 40% to 50% of patients and repeat analysis can improve the rate of detection to 86% with the second tap and 90% with the third tap.^2,12^

The treatment of LMD once diagnosed involves chemotherapy, either administered intrathecally or systemically. Intrathecal chemotherapy most commonly involves the administration of methotrexate and or liposomal cytarabine, often in combination with hydrocortisone.^1,5,13^ Radiation therapy to the meninges can also be administered and may be more effective than intrathecal chemotherapy for immediate relief of symptoms, especially when caused by bulky tumors, as penetration of chemotherapy in these areas may be limited.^14^ Chemotherapeutic agents used for systemic treatment include methotrexate and cytarabine administered in high doses.^1,15^ The overall prognosis of patients diagnosed with LMD remains poor despite the available treatments.^2,5,7^ The goal of treatment is to provide symptomatic relief for patients. Death usually results from neurological deficits, with median survival ranging between 4 and 6 weeks without treatment.^10^

## Conclusion

Leptomeningeal disease can occur rarely in the setting of advanced solid malignancies. It is accepted that in a patient with active malignancy and new neurologic signs or symptoms, LMD should be considered and radiographic imaging of the brain/spine and examination of the CSF performed. However, the symptoms and presentation can often be mild, making early diagnosis difficult. Early diagnosis of LMD is paramount for prompt initiation of chemotherapy and or radiation therapy. Our case exemplifies the fact that solid tumors may manifest with LMD even in the absence of systemic disease. In cases where there is a reliable serum tumor marker, rising tumor markers in the absence of systemic disease may prompt investigation for LMD.
